# Promotive role of USP29-mediated deubiquitination in malignant proliferation of colorectal cancer cells via the KIAA1429/SOX8 axis

**DOI:** 10.17305/bjbms.2022.7930

**Published:** 2023-05-01

**Authors:** Juncai Li, Jianguo Yang, Zhenzhou Chen, Li Liu, Hao Wang, Qican Deng, Yajun Chen, Yong Que, Zhongxue Fu

**Affiliations:** 1 Department of Gastrointestinal Surgery, The First Affiliated Hospital of Chongqing Medical University, Chongqing, China; 2 Department of Surgery, The People’s Hospital of Yubei District of Chongqing City, Chongqing, China; 3 The Third Affiliated Hospital of Chongqing Medical University, Chongqing, China; 4 Department of Surgery, The First Affiliated Hospital of Chengdu Medical College, Chengdu, China; 5 Department of Gastroenterology, People’s Hospital of Chongqing Hechuan, Chongqing, China

**Keywords:** Colorectal cancer (CRC), ubiquitin-specific peptidases 29 (USP29), KIAA1429, SRY-box transcription factor 8 (SOX8), TPD52, malignant proliferation

## Abstract

Colorectal cancer (CRC) is regarded as one of the most prevalent neoplasms worldwide, and ubiquitination and N6-methyladenosine (m6A) modification regulate the outgrowth of multiple cancers. This study attempted to explore the effect of ubiquitin-specific peptidases 29 (USP29) on the malignant proliferation of CRC cells via stabilizing Vir-like m6A methyltransferase associated (VIRMA/KIAA1429). First, upregulations of USP29, KIAA1429, and SRY-box transcription factor 8 (SOX8) were found in CRC tissues and cells through real-time quantitative polymerase chain reaction and Western blotting. After transfection of si-USP29, the proliferation of CRC cells was evaluated by the cell counting kit-8, colony formation, and 5-ethynyl-2’-deoxyuridine assays, and we observed that depletion of USP29 inhibited the proliferation of CRC cells. Co-immunoprecipitation confirmed the binding of USP29 to KIAA1429. Mechanically, USP29 mediated deubiquitination to stabilize the protein levels of KIAA1429, and KIAA1429 promoted the stability of SOX8 mRNA through m6A modification. Moreover, overexpression of KIAA1429 or SOX8 reversed the inhibitory effects of USP29 depletion on CRC cell proliferation. Finally, the xenograft tumor model revealed the promotive role of USP29 in the proliferation of CRC cells in vivo. Altogether, USP29 facilitates the malignant proliferation of CRC cells via upregulating the KIAA1429/SOX8 axis.

## Introduction

Colorectal cancer (CRC) is a fatal solid malignancy prevalent in females as the second most frequently diagnosed cancer and in males as the third most, accounting for ~10% of all cancer cases and cancer-related deaths across the world [1]. Currently, despite the advance in standard treatment regimens for CRC, the battle against CRC is still uphill due to its propensities for drug resistance, recurrence, and the side effects imposed by these regiments [2]. In contrast to the early stages, CRC is strongly correlated to the low long-term survival rates of CRC patients in advanced stages [3]. Hence, discovering novel biomarkers is paramount for the prediction and treatment for CRC.

Ubiquitination, mainly activated by enzymes E1, E2, and E3, is key to post-translational modification of proteins and the regulation of several eukaryotic signaling pathways, thus affecting a broad range of pathophysiological states and disorders [4, 5]. As the largest subgroup of deubiquitinating enzymes (DUBs), ubiquitin-specific proteases (USPs) are potent to counter the processes of ubiquitin signaling on proteins [6]. Notably, USP1 downregulation enhances the sensitivity of CRC cells to chemotherapeutic agents [7], and USP21 is shown to accelerate metastasis of CRC via mediating deubiquitination of fos-related-antigen-1 [8]. Most importantly, USP29 exhibits proliferative and carcinogenic roles in CRC [9], suggesting the potential of USP29 to be a favorable target for treating CRC.

USP29 is potent to interact with N6-methyladenosine (m6A) modification [10]. m6A is a well-studied RNA modification catalyzed by methyltransferases and reversed by demethylases and possesses regulatory functions in physiological and pathological states of eukaryotes [11]. In our follow-up experiments, Vir-like m6A methyltransferase associated (VIRMA/KIAA1429) was found to be regulated by USP29 mediated deubiquitination. KIAA1429 is deemed as a member of the group of m6A methyltransferase complexes and plays a role in recruiting and connecting the catalytic core components, such as METTL3 and METTL14, and RNA substrates, and therefore guides m6A to act at specific regions [12]. Increasing studies have demonstrated the promotive role of KIAA1429 in the development of human malignancies [13, 14]. Besides, KIAA1429 accelerates aerobic glycolysis in CRC and promotes the outgrowth of CRC [15, 16]. However, whether USP29 has an impact on CRC by regulating KIAA1429 has yet to be investigated.

SRY-box transcription factor 8 (SOX8), a member of the SOX-E group in the SOXs family, is found to be widely expressed in both embryonic and mature brain tissues and participates in cancer progression [17]. SOX8 is abundantly expressed in multiple cancers, such as tongue squamous cell cancer and CRC, and is associated with poor prognostic outcomes [18, 19]. Moreover, the follow-up prediction showed the interaction between KIAA1429 and the mRNA of SOX8. Therefore, we questioned that whether USP29-mediated deubiquitination facilitates the proliferation of CRC cells via the KIAA1429/SOX8 axis and we attempted to find novel biomarkers for CRC treatment by clarifying this mechanism.

## Materials and methods

### Tissue collection

The cancerous and para-cancerous tissues from 90 patients with CRC who were admitted to The First Affiliated Hospital of Chongqing Medical University were collected. All patients were confirmed to be preoperatively untreated with chemotherapy or radiotherapy. All cancerous specimens were confirmed as CRC specimens by at least two pathologists. As for the para-cancerous tissue specimens, the normal colorectal mucosal epithelial tissues at a distance of 5 cm from the tumor margin and were extracted and used as the control for cancerous specimens. After tissue collection, all specimens were instantly stored in liquid nitrogen. We conducted our experimental schemes strictly in compliance with the Declaration of Helsinki and these schemes were authorized by the Ethics Committee of The First Affiliated Hospital of Chongqing Medical University, and all CRC patients signed the informed consent form.

### Cell culture

Human normal colonic epithelial cells (FHC) and CRC cells (SW620, HCT116, HCT8, and RKO) provided by Cell Bank of Chinese Academy of Sciences (Shanghai, China) were inoculated in a Dulbecco’s modified Eagle’s medium supplemented with 10% fetal calf serum (Gibco, Grand Island, NY, USA) and 100 U/mL penicillin/streptomycin at 37 ^∘^C with 5% CO_2_.

### Cell treatment

pcDNA3.1-KIAA1429 (KIAA1429), pcDNA3.1-SOX8 (SOX8), and pcDNA3.1-empty vector (NC) were obtained from GenePharma (Shanghai, China) and small interfering (si)-RNAs si-USP29#1, si-USP29#2, si-USP29#3, and NC siRNA (si-NC) were synthesized by Ribo Biotechnology (Guangzhou, Guangdong, China). When SW620 or HCT116 cells reached confluence of 60%–70%, cells were immediately transfected with the above expression vectors using Lipofectamine 3000 (Invitrogen, Carlsbad, CA, USA). The follow-up experiments were performed 48 h after transfection.

### Cell counting kit-8 (CCK-8) analysis

Analysis of cell proliferation was conducted with the help of the CCK-8 kit (Keygen Biotech Co., Ltd., Nanjing, Jiangsu, China) following the instructions. SW620 or HCT116 cells that underwent transfection were seeded in 96-well plates at a density of 1 × 10^4^ cells/well, and each well was added with 10 µL of CCK-8 reagent at 0, 24, 48, and 72 h after transfection. Thereafter, cells were placed back into the incubator and continually incubated for 2 h, and the absorbance at a wavelength of 450 nm was examined using a microplate reader (Bio-Rad, Hercules, CA, USA).

### Colony formation assay

Transfected SW620 or HCT116 cells were seeded in 6-well plates at a density of 1 × 10^3^ cells/well and cultured at 37 ^∘^C for another two weeks, and then SW620 or HCT116 cells were fixed with 4% paraformaldehyde and stained with 0.1% (w/v) crystal violet for 30 min. Counting of cell colonies was conducted using the Image-Pro Plus 5.0 software, and a colony consisting of at least 50 cells was considered available.

### 5-ethynyl-2’-deoxyuridine (EdU)

SW620 or HCT116 cells in logarithmic phase were seeded in 96-well plates at a density of 1 × 10^5^ cells/well and cultured to normal growth phase, and then, based on the requirements of the producer (Ribo Biotechnology), cells were labeled with EdU, stained with Apollo and DNA. Lastly, a fluorescence microscope was adopted to observe the cells.

### N6-methyladenosin (m6A) detection

Following the producer’s instructions, the total RNA in CRC cells was extracted using the TRIzol reagent (Invitrogen), and RNA quality was analyzed using the Nano-Drop 3000. Then, the m6A RNA methylation quantification kit (ab185912, Abcam, Cambridge, MA, USA) was employed to quantify m6A in the total of RNA and the levels of m6A were colorimetrically quantified based on the absorbance at 450 nm of each well.

### RNA immunoprecipitation (RIP) assay

CRC cells were lysed on ice using the lysis buffer containing protease plus ribonuclease inhibitor, after which the cell lysates were incubated with protein A/G beads covered with KIAA1429-specific antibody (ab271136, Abcam) or IgG antibody (ab133470, Abcam) at 4 ^∘^C overnight with rotation. Then, the RIP assay was performed using the Magna RIP RNA-binding protein IP kit (Merck Millipore, Billerica, MA, USA). The association between KIAA1429 and SOX8 mRNA was validated using reverse transcription quantitative-polymerase chain reaction (RT-qPCR).

### Methylated RNA Immunoprecipitation (Me-RIP) assay

Overall, the total RNA isolated from CRC cells was incubated with m6A antibody (ab208577, Abcam) or IgG antibody-conjugated (ab133470, Abcam) protein A/G beads in IP buffer at 4 ^∘^C overnight, and then Me-RIP assay was performed based on the instructions provided by the producer. After elution and purification of the total RNA using the eluting buffer, the enrichment of m6A-precipitated mRNA was detected using RT-qPCR.

### RNA stability assay

To identify the half-life of SOX8 mRNA in CRC cells, cells were added with actinomycin-D (Act-D; 5 mM; Sigma, St. Louis, MO, USA) and placed for 0, 3, and 6 h, respectively. Subsequently, the total RNA was collected at the appointed time, and the remaining SOX8 mRNA was examined.

### Co-immunoprecipitation (Co-IP) assay

The total protein in CRC cells was yielded using the radioimmunoprecipitation assay (RIPA) buffer and centrifuged at 12,000 *g* at 4 ^∘^C to obtain the supernatant. Then, the supernatant underwent incubation with protein A+G agarose beads (Beyotime, Shanghai, China) for 4 h at 4 ^∘^C, and in the meantime, USP29 antibody (PA5-104441, 1:1000, ThermoFisher Scientific, Waltham, MA, USA) or IgG antibody (ab133470, Abcam) was added as the control. Finally, the immunoprecipitated protein was analyzed via Western blotting.

### Ubiquitination detection

To detect the ubiquitination levels of KIAA1429, MG132 (Sigma) at a final concentration of 20 µM was used to treat CRC cells for 8 h to block proteasome degradation. CRC cells were lysed using the RIPA buffer, immunoprecipitated with antibody anti-KIAA1429 (ab271136, Abcam), and then immunoblotted with antibody anti-ubiquitin (ab134953, Abcam).

### Animal experiments

Animal experiments were authorized by the Ethics Committee of The First Affiliated Hospital of Chongqing Medical University, and the use, care, and handling procedures for all animals conformed to the Guide for the Care and Use of Laboratory Animals posted by the National Institutes of Health [20]. BALB/c nude mice (6–8 weeks; 18–20 g; Beijing Vital River Laboratory Animal Technology Co., Ltd., Beijing, China; SYXK (Beijing) 2017-0033) were housed in standard animal chambers, under 12 h-light/dark alternations, with free access to food and water. Based on the producer’s protocols (Biotechnology), SW620 cells were seeded in 6-well plates and infected with USP29-knockdown lentivirus (sh-USP29) or the negative control (sh-NC), respectively, and cells were treated with 4 µg/mL of puromycin (Invitrogen) for two weeks to screen stable knockout cells. Afterward, each mouse was subcutaneously injected via the axilla with stable infected SW620 cells (*N* ═ 5 × 10^6^ cells). Tumors in mice were examined every 7 days, with tumor width (W) and length (L) recorded to assess tumor volume (V): V ═ (W^2^ × L)/2. On the 28th day after surgery, mice were subjected to euthanasia via an intraperitoneal injection of ≥100 mg/kg pentobarbital sodium to resect tumors, and the tumor weight was recorded.

### Immunohistochemistry

Tumor tissues were fixed with 4% paraformaldehyde, embedded in paraffine, dewaxed, and rehydrated, and then were made into 4 µm sections and incubated in 3% H_2_O_2_. Sections were immersed in Tris-EDTA solution containing 0.05% Tween-20 for 30 min at 95 ^∘^C. After rinsing with phosphate-buffered saline (PBS), sections were incubated with PBS solution containing 4% dry milk and 0.3% goat serum for 20 min, and with anti-Ki67 antibody (ab15580, Abcam) overnight. Sections then were incubated with secondary antibody IgG (ab6721, Abcam). Nuclei were counterstained using hematoxylin in slides, dehydrated, and sealed with neutral glue. Sections were examined under a microscope (CKX51, Olympus, Tokyo, Japan).

### RT-qPCR

The total RNA in collected tissues and cells were separated using the TRIzol reagent and synthesized into the complementary RNA via the script cDNA synthesis Kit (Bio-Rad). PCR quantification was performed using the SYBR Premix Ex Taq II (TaKaRa, Shiga, Japan) on CFX96 real-time PCR detection System (Bio-Rad). Gene data were calculated using the 2^−ΔΔCt^ method [21] and normalized to GAPDH. Primer sequences are presented in Table 1.

**Table 1:** PCR primers

**Genes**	**Sequences (5’-3’)**
*USP29*	F: TGCTTGGTTGCTGGTGAAGA
	R: CTGCTCAAGGGAAGAGGTGG
*KIAA1429*	F: CGAGCGCTGAGCAAAGTTCT
	R: TGGGGGTATGACTCGGACTT
*SOX8*	F: AGAAGGACCACCCCGACTAC
	R: AGCCCTGCTTCAGCCTTGTA
*GAPDH*	F: GATGCTGGCGCTGAGTACG
	R: GCTAAGCAGTTGGTGGTGC

USP29: Ubiquitin-specific peptidases 29; SOX8: SRY-box transcription factor 8.

### Western blotting

Cells were incubated with 1% phenylmethanesulfonyl fluoride-contained RIPA buffer (Sigma) on ice for 30 min to separate protein, and the protein concentrations were quantified using a bicinchoninic acid protein detection kit (Beyotime). Protein was subjected to sodium dodecyl sulfate-polyacrylamide gel-electrophoresis and transferred onto polyvinylidene fluoride membranes, followed by blockade using 5% defatted milk for 2 h at room temperature. Later, membranes were incubated with primary antibodies against USP29 (PA5-104441, 1:1000, ThermoFisher Scientific), KIAA1429 (ab271136, 1:1000, Abcam, 202 kDa), and GAPDH (ab181602, 1:10000, Abcam) overnight at 4 ^∘^C and with secondary antibody (1:2000, ab205718, Abcam) for 1 h at room temperature. The intensity of the bands was identified using the chemiluminescence system (Bio-Rad) and analyzed using ImageJ software.

**Figure 1.**
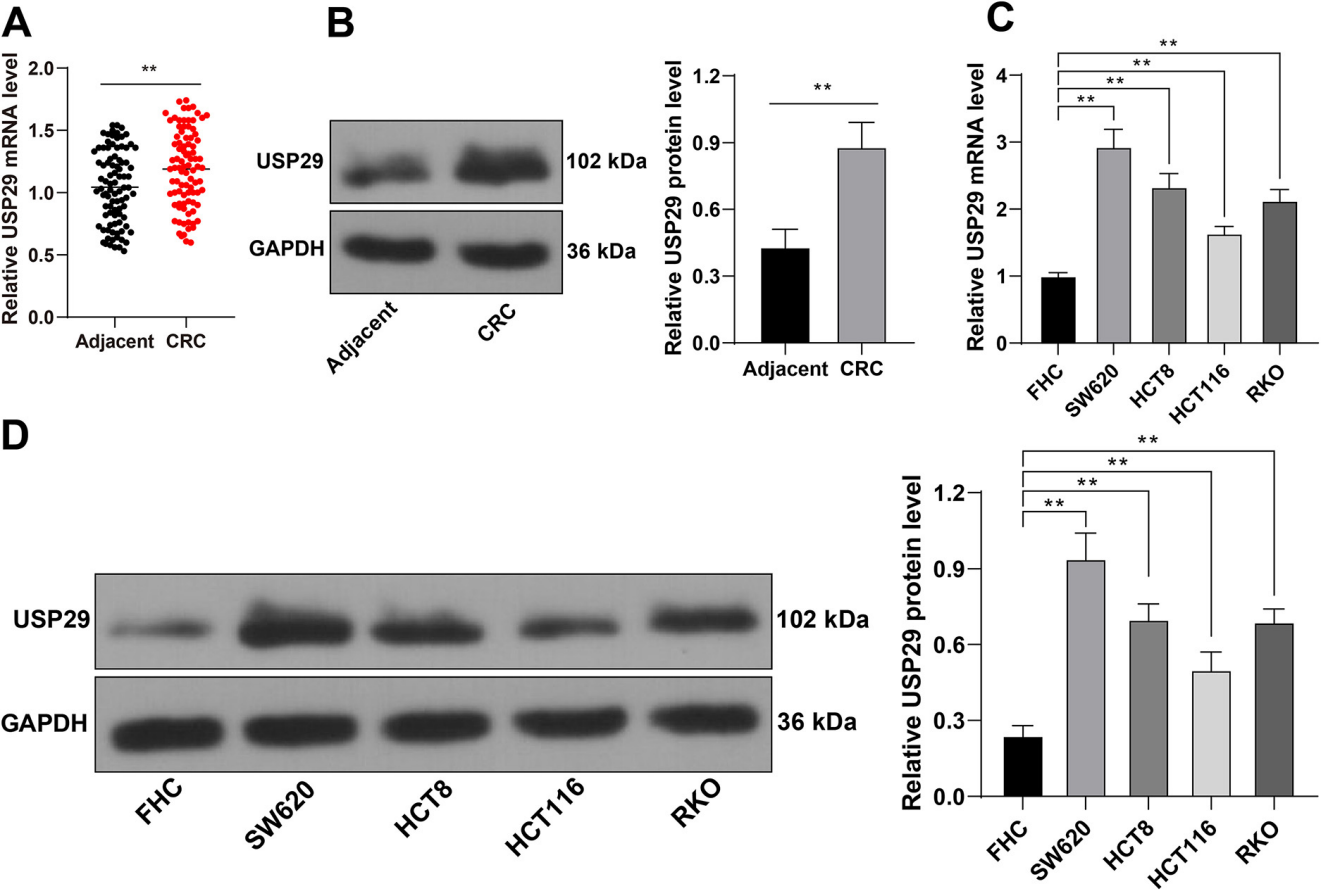
**USP29 is robustly expressed in CRC.** (A) and (B): The expression levels of USP29 in CRC tissues and para-cancerous tissues (*N* ═ 90) were detected via RT-qPCR and Western blotting; (C) and (D): The expression levels of USP29 in each cell line were detected via RT-qPCR and Western blotting. Cell experiments were repeated three times independently. Data in images (B)–(D) were represented by mean ± standard deviation. The *t*-test was employed to analyze data in images (A) and (B); one-way ANOVA was appointed to analyze data in images (C) and (D), followed by Tukey’s post hoc test; **p < 0.01. USP29: Ubiquitin-specific peptidases 29; CRC: Colorectal cancer; RT-qPCR: Reverse transcription quantitative-polymerase chain reaction; ANOVA: Analysis of variance.

### Ethical statement

We conducted our experimental schemes strictly in compliance with the Declaration of Helsinki and the scheme was authorized by the Ethics Committee of The First Affiliated Hospital of Chongqing Medical University (approval number 2022-K480), and all CRC patients signed the informed consent form. Animal experiments were authorized by the Ethics Committee of The First Affiliated Hospital of Chongqing Medical University (approval number 2022-K480), and the use, care, and handling procedures for all animals conformed to the Guide for the Care and Use of Laboratory Animals posted by the National Institutes of Health [20].

### Statistical analysis

SPSS 21.0 software (IBM SPSS Statistics, Chicago, IL, USA) and GraphPad Prism 8.0 software (GraphPad Software Inc., San Diego, CA, USA) were adopted for statistical analyses and graphing. Measurement data were verified to be normally distributed and uniform in variance. The count data were expressed as the number of cases, and the comparison between groups was analyzed by the Chi-square test. Measurement data comparisons between two groups were examined using the *t*-test, and comparisons among multiple groups were examined using one-way or two-way analysis of variance (ANOVA), followed by Tukey’s post hoc test. Values of *p* < 0.05 indicated a statistical significance and *p* < 0.01 indicated a highly statistical significance.

## Results

### USP29 is robustly expressed in CRC

Our detections revealed the higher expression levels of USP29 in CRC tissues compared to para-cancerous tissues (*p* < 0.01, Figure 1A and 1B). CRC patients were divided into the low group and the high group with the median value of USP29 expression levels as the critical threshold. Then, the correlation between the expression levels of USP29 and the clinical characteristics of CRC patients was analyzed, and it was found that USP29 expression level was correlated to tumor size, lymph node metastasis, and TNM stage of CRC patients (Table 2). Afterward, the expression levels of USP29 in cultured cells were also detected, and the results showed that USP29 was increased in CRC cells (SW620, HCT8, HCT116, and RKO) (*p* < 0.01, Figure 1C and 1D). To probe the possible effect of USP29 on malignant proliferation of CRC cells, we chose SW620 cells with relatively high expression of USP29 and HCT116 cells with relatively low expression of USP29 for the subsequent experiments.

**Table 2:** Correlation between the expression levels of USP29 and the clinicopathological features of patients with CRC

**Characteristics**	**Number** **(*N* ═ 90)**	**USP29**		***p*-value**
		**Low (*N* ═ 46)**	**High (*N* ═ 44)**	
*Sex*				0.666
Male	43	23	20	
Female	47	23	24	
*Age, years*				0.686
<60	41	20	21	
≥60	49	26	23	
*Location*				0.144
Colon	50	29	21	
Rectal	40	17	23	
*Tumor size*				**0.013**
<5 cm	32	22	10	
≥5 cm	58	24	34	
*Lymph node metastasis*				**0.011**
Negative	30	21	9	
Positive	60	25	35	
*TNM stage*				**0.008**
I-II	35	24	11	
III-IV	55	22	33	

Comparisons between the low group and the high group were analyzed using the Chi-square test. Bold indicates *p* < 0.05. TNM: Tumor node metastasis; USP29: Ubiquitin-specific peptidases 29; CRC: Colorectal cancer.

### Silencing USP29 hampers malignant proliferation of CRC cells

**Figure 2.**
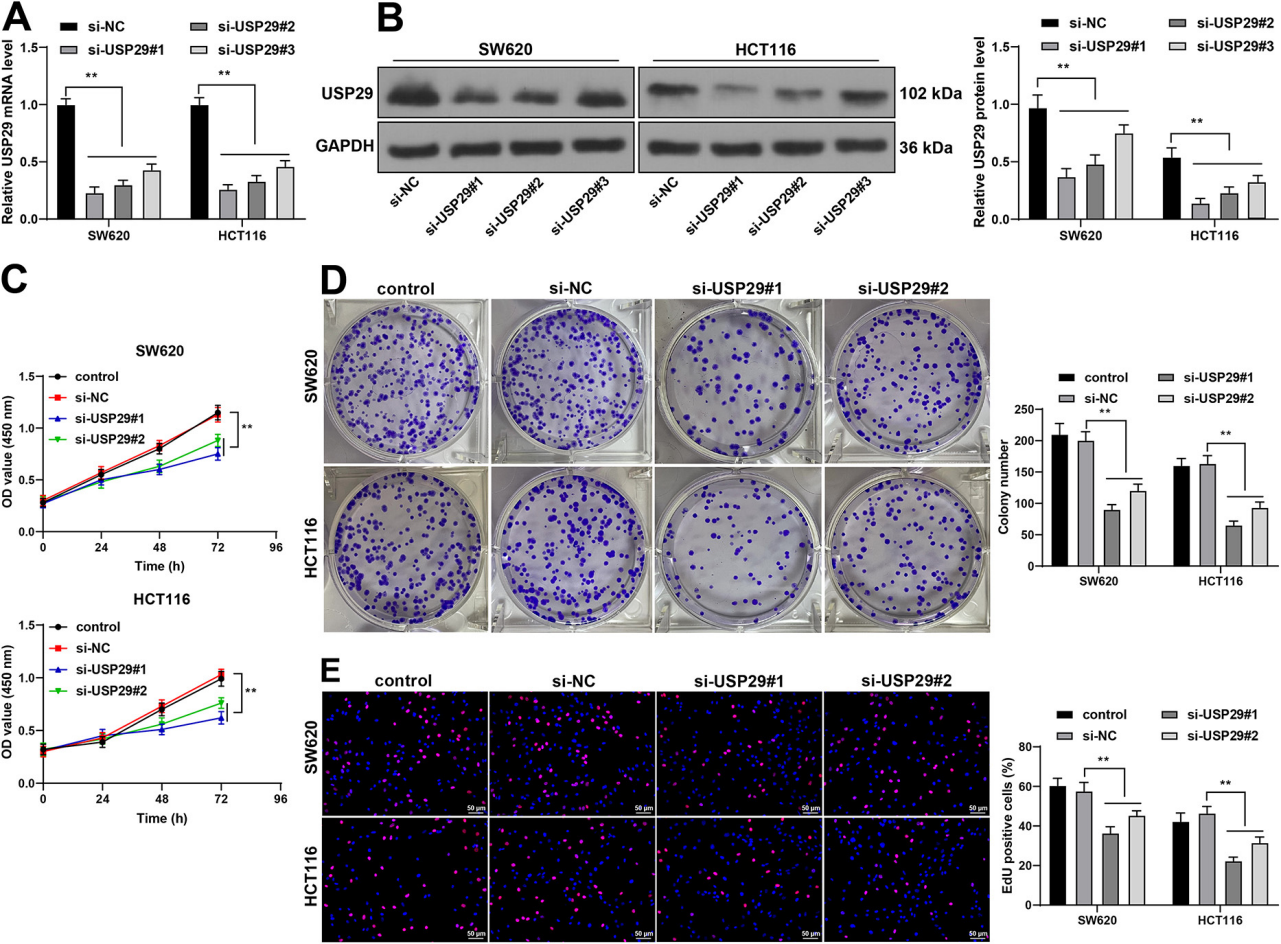
**Silencing USP29 hampers malignant proliferation of CRC cells.** SW620 and HCT116 cells were transfected with USP29 siRNA (si-USP29), and NC siRNA (si-NC) was set as the control. (A) and (B): The expression levels of USP29 in SW620 and HCT116 cells were detected via RT-qPCR and Western blotting; (C)–(E): The proliferation of SW620 and HCT116 cells was determined via the CCK-8, colony formation, and EdU assays. Cell experiments were repeated three times independently. Data are represented by mean ± standard deviation. Two-way ANOVA was used to analyze data in images A–E, followed by Tukey’s post hoc test; **p < 0.01. USP29: Ubiquitin-specific peptidases 29; RT-qPCR: Reverse transcription quantitative-polymerase chain reaction; ANOVA: Analysis of variance; CRC: Colorectal cancer.

The expression levels of USP29 in SW620 and HCT116 cells were downregulated via the transfection of USP29 siRNA (si-USP29) (*p* < 0.01, Figure 2A and 2B), and si-USP29#1 and si-USP29#2 were found to have better intervention efficiency, and, as a result, were chosen for the follow-up experiments. Our results revealed that silencing USP29 diminished SW620 and HCT116 cell viability (*p* < 0.01, Figure 2C), and reduced the number of colonies and EdU-positive cells (*p* < 0.01, Figure 2D and 2E), suggesting that USP29 downregulation suppressed malignant proliferation of CRC cells.

### USP29 stabilizes the protein levels of KIAA1429 via deubiquitination

**Figure 3.**
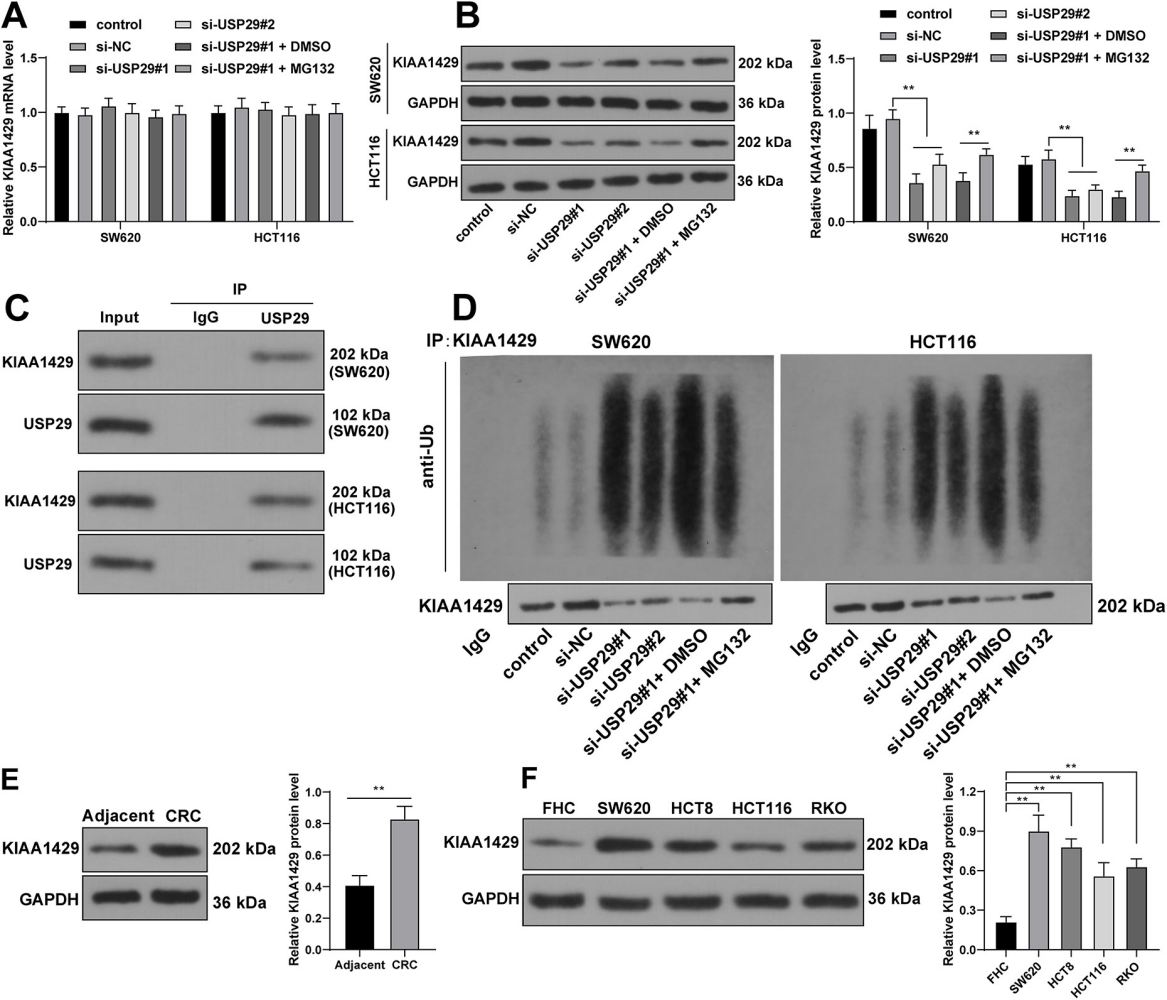
**USP29 stabilizes the protein levels of KIAA1429 via deubiquitination.** (A) and (B): The expression levels of KIAA1429 in CRC cells were examined via RT-qPCR and Western blotting; (C) The interaction between USP29 and KIAA1429 in CRC cells was detected via the Co-IP assay; (D) The ubiquitination levels of KIAA1429 in CRC cells were examined via Western blotting and ubiquitination assays; (E) and (F): The protein levels of KIAA1429 in tissues (*N* ═ 90) and cell lines were measured via Western blotting. Cell experiments were repeated three times independently. Data are represented by mean ± standard deviation. The *t*-test was employed to analyze data in image (E); two-way ANOVA was used to analyze data in images (A) and (B); one-way ANOVA was employed to analyze data in image (F), followed by Tukey’s post hoc test; **p < 0.01. USP29: Ubiquitin-specific peptidases 29; CRC: Colorectal cancer; RT-qPCR: Reverse transcription quantitative-polymerase chain reaction; Co-IP: Co-immunoprecipitation; ANOVA: Analysis of variance.

USP29 is capable of protecting its downstream genes from ubiquitin-mediated proteasome degradation to stabilize their protein levels [22]. KIAA1429 is reported to be expressed at high levels in colonic adenocarcinoma [23]. We speculated that KIAA1429 overexpression in CRC may be related to deubiquitination mediated by USP29. In this connection, KIAA1429 expression levels after the intervention of USP29 were detected and the results showed that though silencing USP29 had no effect on the mRNA levels of KIAA1429 (*p* > 0.05, Figure 3A), it declined the protein levels of KIAA1429 (*p* < 0.01, Figure 3B). Experimental results also revealed that USP29 bound to KIAA1429 and silencing USP29 increased the ubiquitination levels of KIAA1429 (Figure 3C and 3D). Additionally, MG132 (a proteasome inhibitor) was added to SW620 and HCT116 cells transfected with si-USP29#1 to downregulate KIAA1429 ubiquitination levels (*p* < 0.01, Figure 3D), after which the mRNA levels of KIAA1429 had no change (*p* > 0.05, Figure 3A), while the protein levels of KIAA1429 were elevated (*p* < 0.01, Figure 3B). Moreover, our subsequent detections revealed that protein levels of KIAA1429 were indeed increased in CRC tissues and cells (*p* < 0.01, Figure 3E–F). Together, these data indicated that USP29-mediated deubiquitination stabilized KIAA1429 protein levels in CRC cells.

### KIAA1429 upregulation attenuates the inhibitory role of silencing USP29 in malignant proliferation of CRC cells

**Figure 4.**
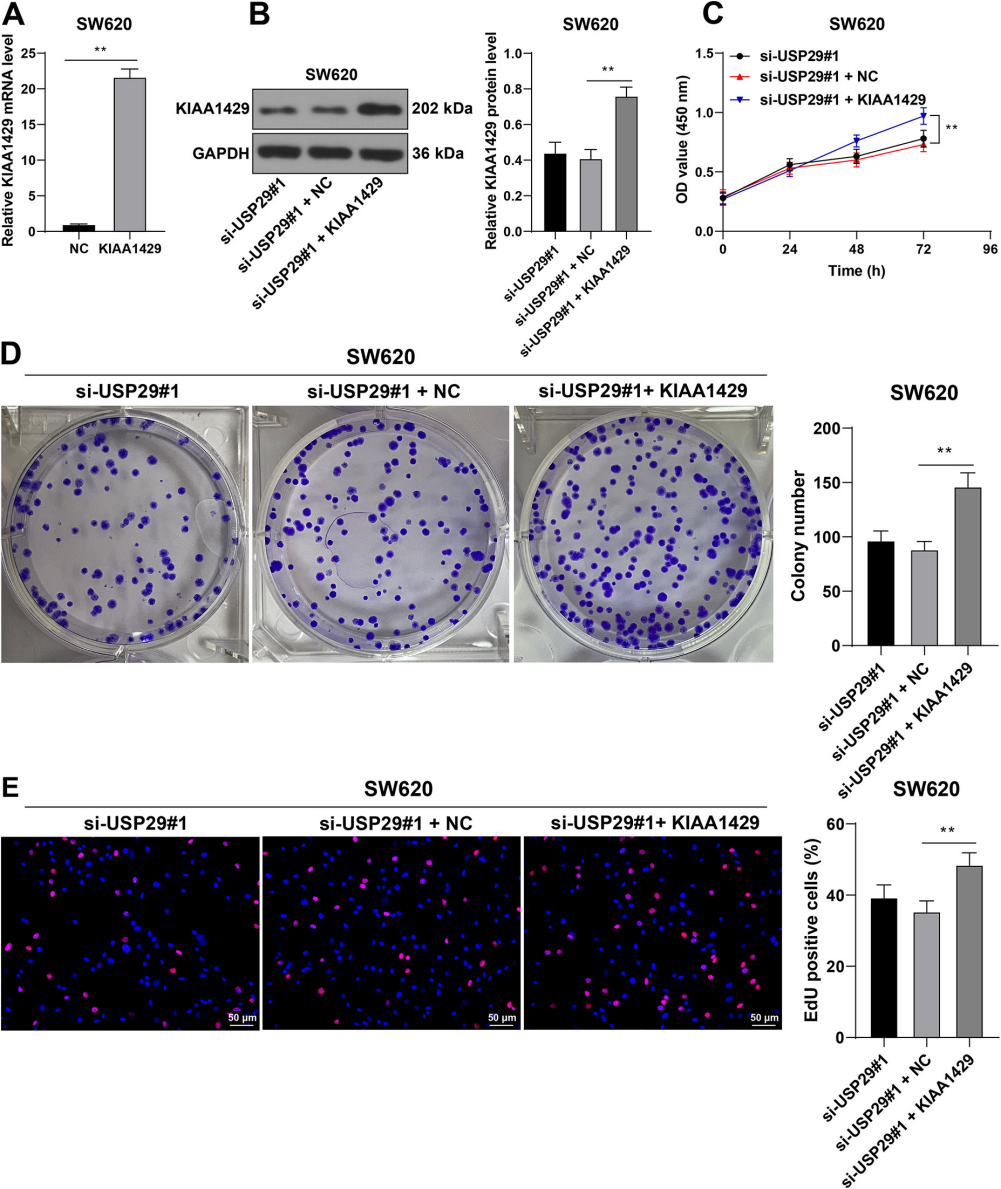
**KIAA1429 upregulation attenuates the inhibition of silencing USP29 on malignant proliferation of CRC cells.** SW620 cells were transfected with pcDNA3.1-KIAA1429 (KIAA1429), and pcDNA3.1-empty vector (NC) was set as the negative control. (A) and (B): The expression levels of KIAA1429 in SW620 cells were detected via RT-qPCR and Western blotting; (C)–(E): The proliferation of SW620 cells was measured via the CCK-8, colony formation, and EdU assays. Cell experiments were repeated three times independently. Data were represented by mean ± standard deviation. The *t*-test was employed to analyze data in image (A); two-way ANOVA was used to analyze data in image (C); one-way ANOVA was employed to analyze data in images (B), (D), and (E), followed by Tukey’s post hoc test; **p < 0.01. USP29: Ubiquitin-specific peptidases 29; CRC: Colorectal cancer; RT-qPCR: Reverse transcription quantitative-polymerase chain reaction; ANOVA: Analysis of variance; Co-IP: Co-immunoprecipitation; EdU: 5-ethynyl-2’-deoxyuridine.

Next, KIAA1429 was upregulated in SW620 cells via the transfection of pcDNA3.1-KIAA1429 (KIAA1429), followed by combination treatment with si-USP29#1 (*p* < 0.01, Figure 4A and 4B), to validate the role of KIAA1429 in malignant proliferation of CRC cells. Compared with the si-USP29#1 + NC group, KIAA1429 upregulation enhanced SW620 cell proliferation (*p* < 0.01, Figure 4C– 4E), which indicated that KIAA1429 upregulation attenuated the inhibitory role of silencing USP29 in malignant proliferation of CRC cells.

### KIAA1429 improves the mRNA stability of SOX8 via m6A modification

**Figure 5.**
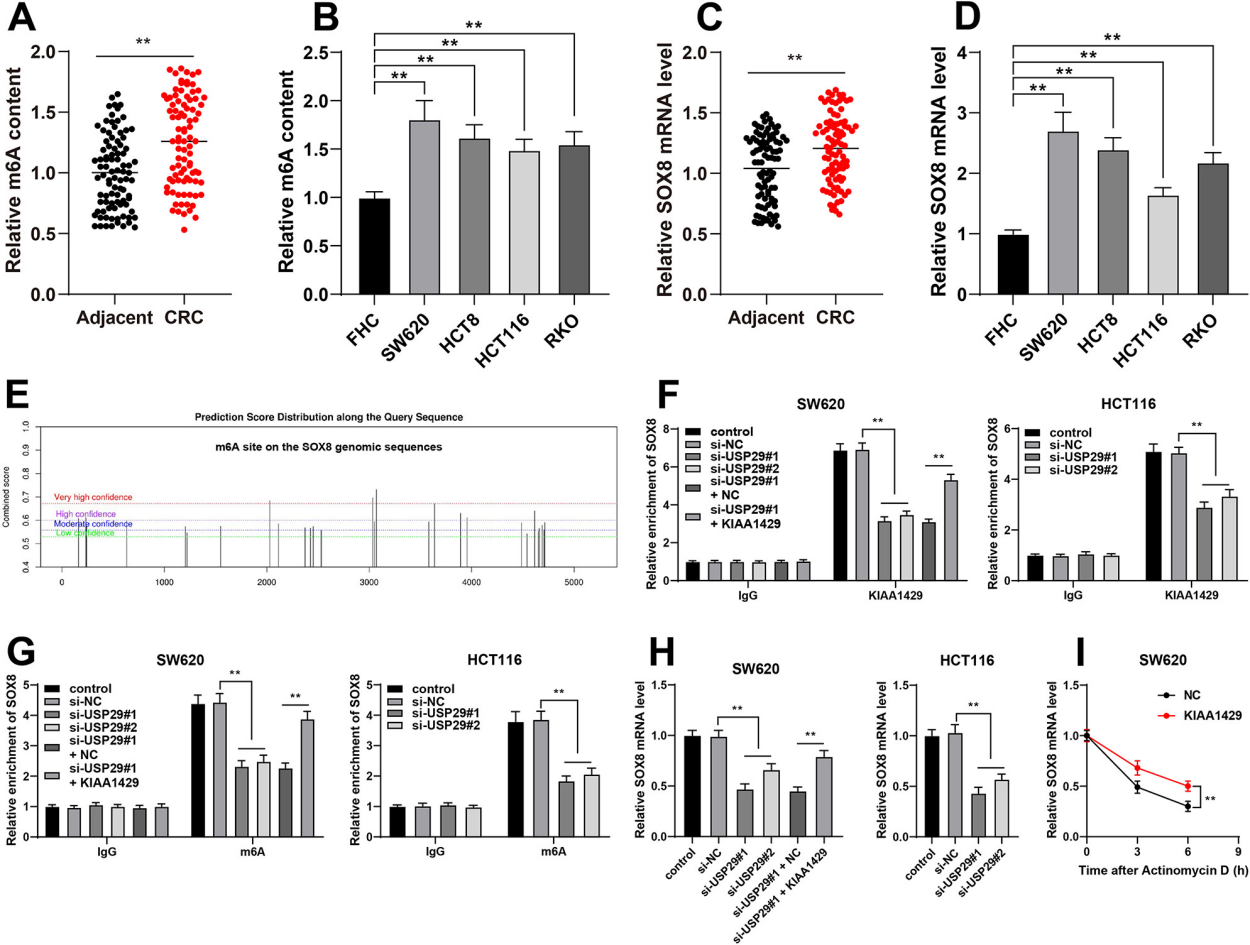
**KIAA1429 improves the mRNA stability of SOX8 via m6A modification.** (A) and (B): The contents of m6A in tissues (*N* ═ 90) and cell lines were analyzed via the m6A quantification detection; (C) and (D): The mRNA levels of SOX8 in tissues (*N* ═ 90) and cell lines were examined via RT-qPCR; (E): The SRAMP website was used to predict the potential m6A modification sites in the SOX8 genome; (F) and (G): The enrichment of KIAA1429 or m6A in SOX8 were determined via the RIP and Me-RIP assays; (H): The mRNA levels of SOX8 in intervened CRC cell lines were detected via RT-qPCR; (I): The mRNA stability of SOX8 after actinomycin-D treatment. Cell experiments were repeated three times independently. Data in images (B), (D), and (F)–(I) were represented by mean ± standard deviation. The *t*-test was employed to analyze data in images (A) and (C); two-way ANOVA was used to analyze data in images (F), (G), and (I); one-way ANOVA was employed to analyze data in images (B), (D), and (H), followed by Tukey’s post hoc test; **p < 0.01. SOX8: SRY-box transcription factor 8; m6A: N6-methyladenosine; RT-qPCR: Reverse transcription quantitative-polymerase chain reaction; RIP: RNA immunoprecipitation; Me-RIP: Methylated RNA Immunoprecipitation; ANOVA: Analysis of variance.

KIAA1429 promotes the stability of mRNA via m6A modification [24], and the m6A levels in CRC tissues and cells were notably increased (*p* < 0.01, Figure 5A and 5B). SOX8 is robustly expressed in CRC [18]. Consistently, we also found increased SOX8 mRNA levels in CRC tissues and cells (*p* < 0.01, Figure 5C and 5D). The SRAMP website revealed that there were potential m6A modification sites in the SOX8 genome (Figure 5E). Subsequently, we found that USP29 depletion reduced the enrichment of KIAA1429 or m6A in SOX8 and declined SOX8 mRNA levels in CRC cells, while KIAA1429 overexpression displayed the opposite trends (*p* < 0.01, Figure 5F–5H). Besides, KIAA1429 upregulation improved the mRNA stability of SOX8 in CRC cells (*p* < 0.01, Figure 5I). Collectively, KIAA1429 enhanced SOX8 mRNA stability by mediating m6A modification.

### SOX8 overexpression averts the inhibitory role of silencing USP29 on malignant proliferation of CRC cells

**Figure 6.**
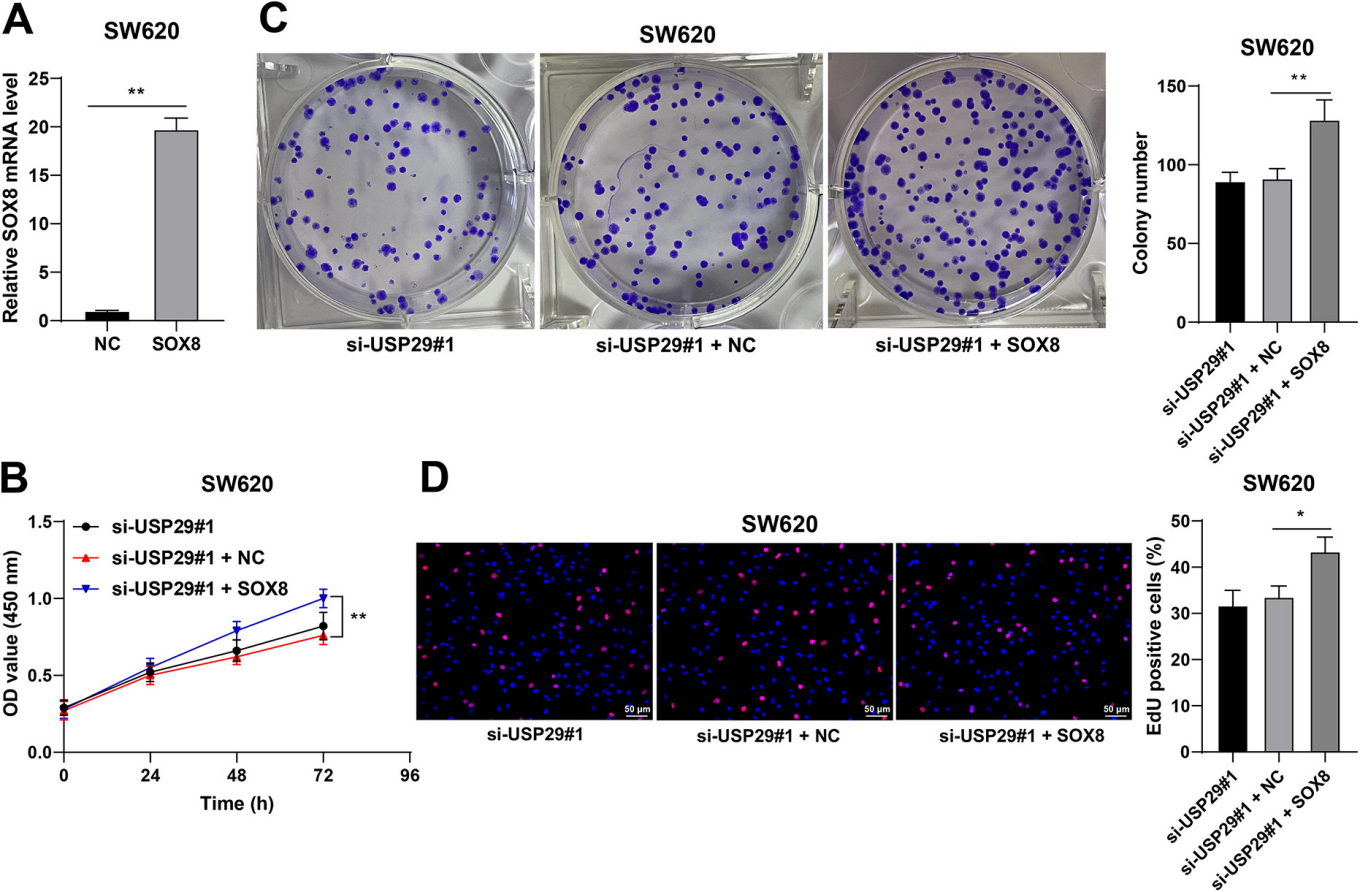
**SOX8 overexpression averts the inhibition of silencing USP29 on malignant proliferation of CRC cells.** SW620 cells were transfected with pcDNA3.1-SOX8 (SOX8), and pcDNA3.1-empty vector (NC) was set as the negative control. (A): The mRNA levels of SOX8 in SW620 cells were detected via RT-qPCR; (B)–(D): The proliferation of SW620 cells was measured via the CCK-8, colony formation, and EdU assays. Cell experiments were repeated three times independently. Data were represented by mean ± standard deviation. The *t*-test was employed to analyze data in image (A); two-way ANOVA was used to analyze data in image (B); one-way ANOVA was employed to analyze data in images (C) and (D), followed by Tukey’s post hoc test; *p < 0.05, **p < 0.01. SOX8: SRY-box transcription factor 8; USP29: Ubiquitin-specific peptidases 29; CRC: Colorectal cancer; RT-qPCR: Reverse transcription quantitative-polymerase chain reaction; CCK-8: Cell counting kit-8; ANOVA: Analysis of variance.

To probe the effect of SOX8 on malignant proliferation of CRC cells, the rescue experiments were conducted: SW620 cells were transfected with pcDNA3.1-SOX8 to upregulate SOX8 expression (*p* < 0.01, Figure 6A) and combined with si-USP29#1. In the si-USP29#1 + SOX8 group, SW620 cell proliferation was augmented (*p* < 0.05, Figure 6B–6D). Hence, the above results suggested that SOX8 overexpression reversed the inhibitory role of silencing USP29 in malignant proliferation of CRC cells.

### Silencing USP29 represses the malignant proliferation of CRC cells in vivo

**Figure 7.**
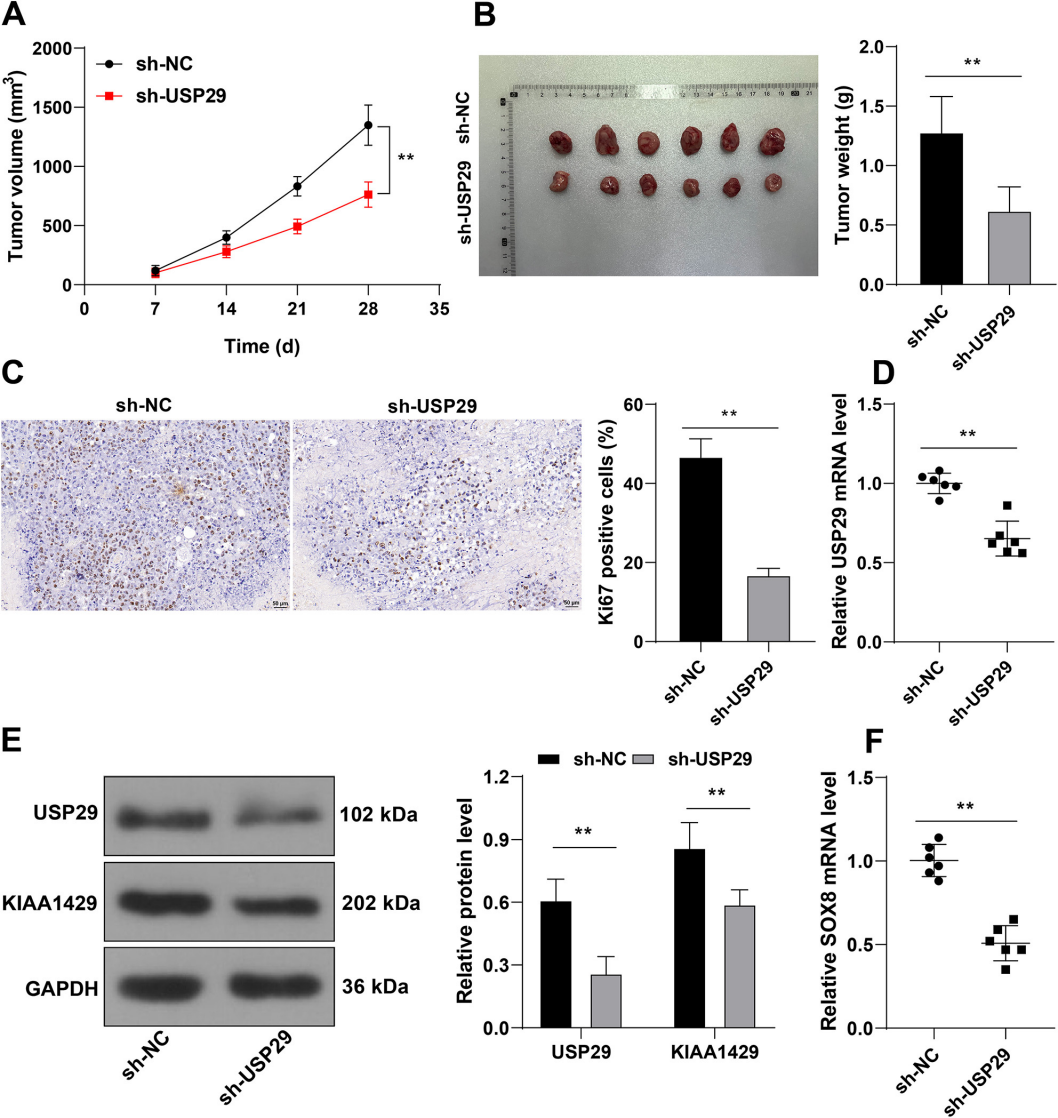
**Silencing USP29 represses malignant proliferation of CRC cells in vivo.** SW620 cells with stable low expression of USP29 were used to establish mouse xenograft tumor models. (A) and (B): The volume and weight of the xenografted tumor were recorded; (C): The positive rates of Ki67 in tumor tissues were detected via immunohistochemistry; (D): The mRNA levels of USP29 in tumor tissues were detected via RT-qPCR; (E): The protein levels of USP29 and KIAA1429 in tumor tissues were examined via Western blotting; (F): The mRNA levels of SOX8 in tumor tissues were determined via RT-qPCR. Cell experiments were repeated three times independently. Data in images (A)–(C) and (E) were represented by mean ± standard deviation. The *t*-test was employed to analyze data in images (B)–(D) and (F); two-way ANOVA was used to analyze data in images (A) and (E), followed by Tukey’s post hoc test; **p < 0.01. USP29: Ubiquitin-specific peptidases 29; CRC: Colorectal cancer; RT-qPCR: Reverse transcription quantitative-polymerase chain reaction; SOX8: SRY-box transcription factor 8; ANOVA: Analysis of variance.

At last, mouse xenograft tumor models were established using SW620 cells with stable low expression of USP29. Silencing USP29 hampered tumor growth, as evidenced by reduced tumor volume and weight (*p* < 0.01, Figure 7A and 7B) and diminished positive rate of Ki67 (*p* < 0.01, Figure 7C). Upon silencing USP29, KIAA1429 protein levels and SOX8 mRNA levels were both decreased (*p* < 0.01, Figure 7D–7F). Together, these data indicated that USP29 downregulation inhibited the proliferation of CRC cells in vivo by decreasing KIAA1429/SOX8 levels.

## Discussion

CRC has been ranked as the third major cause of cancer-related mortality, with an estimation of 1.85 million diagnoses and 850,000 deaths each year, and over 20% of newly diagnosed patients develop metastatic disease at the diagnosis [25]. USPs are cancer-related proteases with the ability to regulate DNA damage repair, chromatin remodeling, and cell cycle in cancers and have become a hot spot in the researches on anti-cancer therapies [26]. From the observations of this study, we revealed the promotive role of USP29 in malignant proliferation of CRC cells via the KIAA1429/SOX8 axis.

Mounting studies have elucidated the multifaceted roles of USP29 in cancers. USP29 blocks Snail degradation and accelerates epithelial-mesenchymal transition (EMT) and cell migration in gastric cancer [27]. Besides, USP29 overexpression augments stemness of lung cancer cells after chemotherapy [28] while USP29 knockdown reduces aerobic glycolysis and attenuates Sorafenib resistance in hepatocellular carcinoma cells [29]. In this present work, we found the correlation between USP29 expression levels and the tumor size, lymph node metastasis, and TNM stage of CRC patients and USP29 upregulation in CRC tissues and cells. Expectedly, the depletion of USP29 effectively limited the proliferation of SW620 and HCT116 cells in vitro, and the xenograft tumor models revealed that USP29 downregulation inhibited the proliferation of CRC cells in vivo. In accordance, Chandrasekaran et al. [9] illustrated that silencing USP29 promotes DNA damage and cell apoptosis while hampers cell cycle, potently limiting the growth of CRC cells both in vivo and in vitro. By and large, these findings confirmed the repressive roles of USP29 depletion in the malignant proliferation of CRC cells.

As an outstanding member of DUBs, USP29 prevents its downstream genes from degradation via deubiquitination and therefore mediates tumorigenesis [22]. USPs are reported to modulate m6A methyltransferase. Existing studies have shown that deubiquitination mediated by USP5 or USP12 enhances METTL3 stabilization [30, 31]. In addition, KIAA1429 is evidenced to alter m6A modification on target genes and thus accelerates progression of gastric cancer [32] and contributes to migration and invasion of hepatocellular cancer [14]. More importantly, Xu et al. [23] detected elevated KIAA1429 in colonic adenocarcinoma. Our follow-up experiments showed that USP29 bound to KIAA1429 and silencing USP29 decreased the protein levels of KIAA1429 without the change in the mRNA levels. This is because silencing USP29 increased the ubiquitination levels of KIAA1429 induced by. Then, vectors overexpressing KIAA1429 were co-transfected with vectors silencing USP29 into SW620 cells. We uncovered that KIAA1429 upregulation boosted the proliferation of SW620 cells. Consistently, previous studies have demonstrated that KIAA1429 overexpression promotes the proliferation and migration of CRC dependent or independent on m6A modification [15, 33]. Besides, KIAA1429 overexpression increases aerobic glycolysis in CRC via regulating HK2 [16]. Overall, our findings indicated that USP29 stabilized KIAA1429 protein levels, and KIAA1429 upregulation counteracted the effects of USP29 depletion on the proliferation of CRC cells.

Dependent on m6A modification, KIAA1429 increases the stability of its downstream genes and thus elevates their expressions in cancers [15, 24]. SOX8 is considered a detrimental prognostic factor for triple-negative breast cancer and tongue squamous cell cancer [19, 34, 35]. Additionally, several studies have highlighted the role of SOX8 in augmenting EMT and drug resistance in cancers [36–38]. In the present study, the mRNA levels of SOX8 were markedly increased in CRC, and USP29 knockdown decreased the enrichment of KIAA1429 in SOX8 and declined SOX8 mRNA levels whilst KIAA1429 upregulation counteracted the above trends and enhanced SOX8 mRNA stability. Subsequently, SOX8 levels in SW620 cells were upregulated, followed by silencing USP29, after which SW620 cell proliferation was promoted. SOX8 activates the HGF/MET axis to diminish the sensitivity of CRC cells to cetuximab [39]. Furthermore, Wang et al. reported that the upregulation of SOX8 is positively correlated with the TNM stage I and II stages and a poor overall survival of CRC patients [18]. In vivo, USP29 knockdown decreased KIAA1429 protein levels and SOX8 mRNA levels, therefore limiting proliferation of CRC cells. Altogether, the above data suggested that KIAA1429 stabilized SOX8 mRNA stability and SOX8 overexpression reversed USP29 knockdown-mediated suppression on proliferation of CRC cells.

## Conclusion

**Figure 8.**
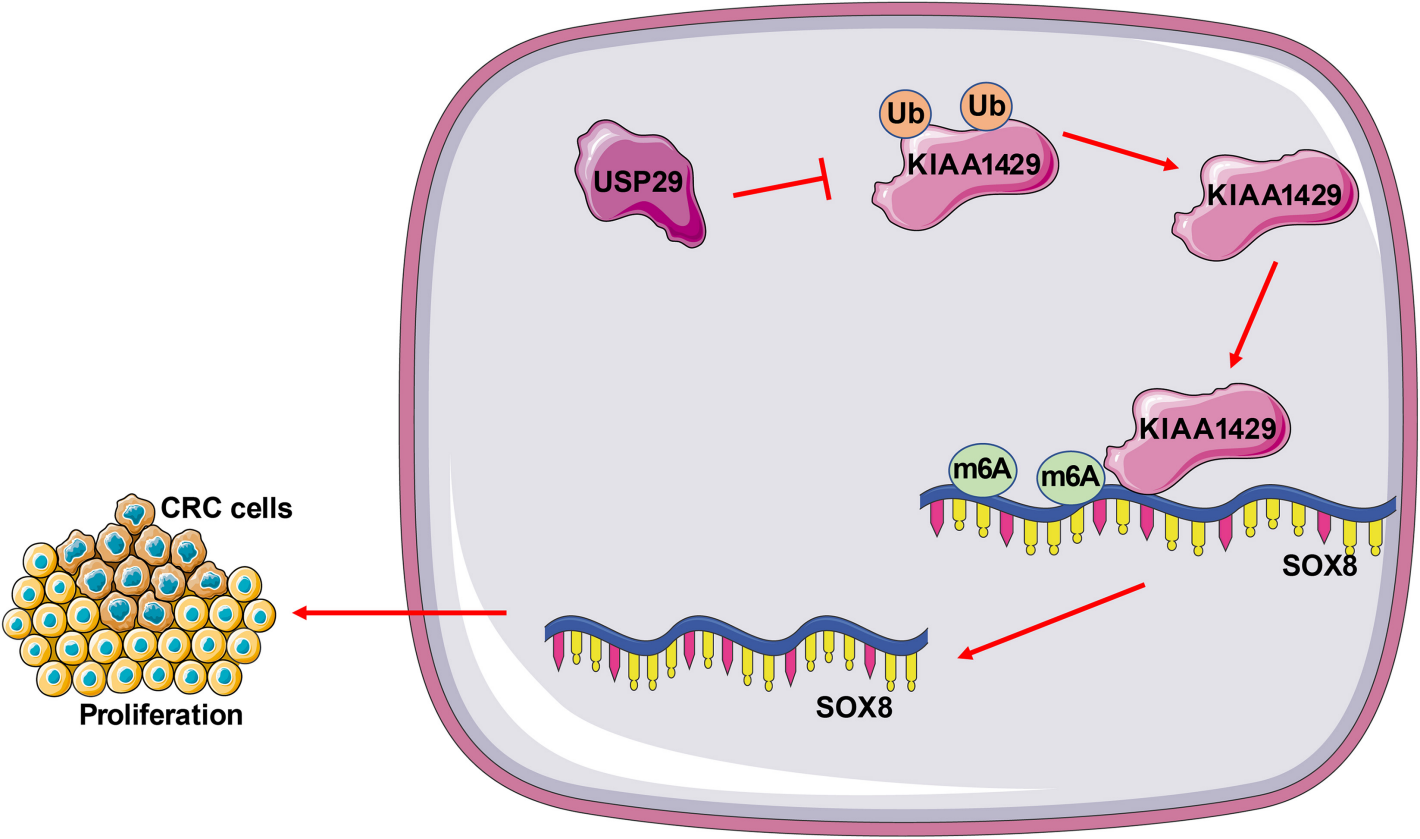
**Mechanism of USP29 in malignant proliferation of CRC cells.** USP29 stabilized KIAA1429 protein levels via deubiquitination, and KIAA1429 enhanced SOX8 mRNA stability by m6A modification, promoting proliferation of CRC cells. USP29: Ubiquitin-specific peptidases 29; CRC: Colorectal cancer; SOX8: SRY-box transcription factor 8; m6A: N6-methyladenosine.

To sum up, our findings initially elucidated that USP29-mediated deubiquitination stabilized KIAA1429 protein levels and further prompted KIAA1429 to increase SOX8 mRNA stability, ultimately facilitating the proliferation of CRC cells (Figure 8), which may help to expand the chances of cure for CRC. Nonetheless, the USP29/KIAA1429/SOX8 mechanism that we studied here is only one of many mechanisms affecting the malignant proliferation of CRC cells. We have yet to probe the role of USP29 in the migration and invasion of CRC cells, or measure SOX8 protein levels and KIAA1429 mRNA levels and evaluate the effect of their changes on CRC cells. Consequently, future study will center on investigating the mechanism of USP29 in the migration and invasion of CRC cells and the upstream of USP29 to offer more theoretical knowledge for CRC treatment.
